# Exercise Therapy Using the Lumbar-Type Hybrid Assistive Limb Ameliorates Locomotive Function after Lumbar Fusion Surgery in an Elderly Patient

**DOI:** 10.1155/2021/1996509

**Published:** 2021-08-22

**Authors:** Yoshihiro Yasunaga, Kousei Miura, Masao Koda, Toru Funayama, Hiroshi Takahashi, Hiroshi Noguchi, Kentaro Mataki, Tomoyuki Asada, Keiji Wada, Yoshiyuki Sankai, Masashi Yamazaki

**Affiliations:** ^1^Department of Orthopaedic Surgery, Faculty of Medicine, University of Tsukuba, 1-1-1 Tennodai, Tsukuba, Ibaraki 305-8575, Japan; ^2^Department of Orthopaedics, Tokyo Women's Medical University, 8-1 Kawada-cho, Shinjuku-ku, Tokyo 162-8666, Japan; ^3^Center for Cybernics Research, University of Tsukuba, 1-1-1 Tennodai, Tsukuba, Ibaraki 305-8575, Japan

## Abstract

The number of elderly people who undergo lumbar fusion surgery (LFS) has been increasing. Postoperative rehabilitation for them can be problematic due to lumbar stiffness. This is the first case report describing exercise therapy using the lumbar-type hybrid assistive limb (HAL) after multiple LFS in an elderly patient. An 83-year-old man underwent LFS at L4-S1. Additional fusion surgery at L2-3 for adjacent segment disease was performed 2 years after the primary surgery. Although the patient's leg pain declined, he had severe locomotive dysfunction at 3 months after his final surgery. He started exercise therapy using the lumbar-type HAL including sit-to-stand training and squat training 4 months after his final surgery. He performed 3 sets of 20 sit-to-stand and 20 squat repetitions with adequate rests in between sets. The HAL training was safely conducted every day for 12 weeks without adverse event. Timed up and go test (TUG), 1-minute sit to stand test (1MSTS), one-leg stand test (OLST), and Berg balance scale (BBS) were assessed as locomotive function measurement. Before HAL therapy, TUG, 1MSTS, OLST, and BBS were 18.1 sec, 20 times, less than 1 sec, and 47, respectively. He could not walk without assistance. After the exercise therapy with the lumbar-type HAL, his locomotive function dramatically improved. TUG, 1MSTS, OLST, and BBS were 12.2 sec, 25 times, 3.9 sec, and 52, respectively. Moreover, the patient could walk 60 meters continuously without assistance. The unique characteristics of the lumbar-type HAL to prevent the lumbar overload and assist the voluntary hip joint motion during exercise therapy may be effective for this patient with lumbar stiffness after LFS. Sit-to-stand training and squat training using the lumbar-type HAL are promising options to improve locomotive function in elderly patients after LFS.

## 1. Introduction

The aging of society has been increasing in recent years in many countries and is a particularly important issue in Japan. Along with an increase in the elderly population, their utilization of lumbar fusion surgery (LFS) has been increasing. A rapid increase in the rate of lumbar fusion surgery among those aged more than 60 years has been reported [1]. The dominant view is that lumbar fusion surgery improves low back pain and leg pain for lumbar degenerative diseases [2, 3]. However, lumbar stiffness after fusion surgery can lead to limitations in activities of daily living (ADL) [4]. In particular, postoperative Oswestry Disability Index (ODI) scores are relatively high among elderly people after lumbar fusion surgery [5]. Postoperative rehabilitation can be useful to resolve this. Various types of rehabilitation after LFS, such as cardiovascular exercise, soft-tissue mobilization, nerve mobilization, motor control, strength training, joint mobilization, and patient education, have been reported [6]. Shariat et al. pointed out that the therapeutic package, including exercise movements and psychological interventions, improved lumbar range of motion (ROM), resulting in greater lumbar flexibility [7]. On the other hand, gradual increase of exercise load is needed for safety in elderly patients [8]. Thus, exercise therapy must not overload the spine in order to be effective for elderly patients.

For this reason, we used a robotic exoskeleton called the hybrid assistive limb (HAL) to perform exercise therapy after LFS. A lower limb type HAL and a single joint HAL have been used for rehabilitation in several diseases. The lumbar-type HAL can suppress the wearer's lumbar motion and assist hip joint motion. By detecting muscle action potentials of the erector spinae muscles through electrodes on the wearer's skin, the lumbar-type HAL can assist voluntary joint motion by reacting to the wearer's intention. To date, it has been reported that the lumbar-type HAL may help healthy volunteers by reducing lumbar overload in physically heavy tasks such as lifting movements, snow-shoveling movements, and simulated patient transfer movements [9–11]. Recent advances in the robotic exoskeleton for rehabilitation have been remarkable. However, rehabilitation using the robotic exoskeleton after LFS has been uncommon. It particularly seems important to attempt to prevent lumbar overload by using robotic assist during postoperative rehabilitation in an aging society. Here, we present a first case where exercise therapy using the lumbar-type HAL following LFS ameliorated locomotive function.

## 2. Case Presentation

### 2.1. Patient

**Figure 1:**
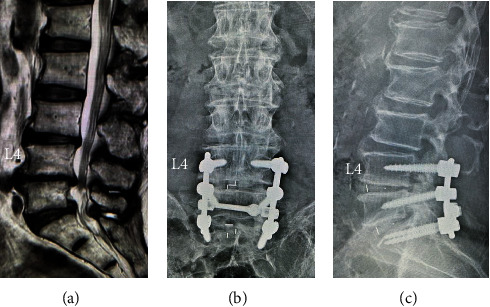
(a) Preoperative sagittal T2-weighed magnetic resonance imaging showing bulging discs and canal stenosis at L4-5 and L5-S1. (b, c) Postoperative radiographs after posterior fusion surgery at L4-S1.

**Figure 2:**
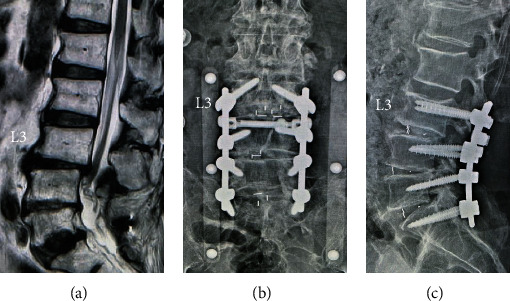
(a) Sagittal T2-weighed magnetic resonance imaging 2 years after primary surgery showing posterior spondylolisthesis, bulging disc, and canal stenosis at L3-4. (b, c) Postoperative radiographs after additional posterior fusion surgery at L3-4.

An 83-year-old man presented with back pain, as well as left leg pain and numbness. He had a history of cervical laminoplasty at C3-7 for cervical spondylotic myelopathy 4 years earlier and prostatic hypertrophy treated with medication. Magnetic resonance imaging (MRI) revealed bulging discs and canal stenosis at L4-5 and L5-S1. He underwent instrumented lumbar fusion surgery at L4-S1 after conservative therapy did not result in any improvement (Figure 1). His symptoms improved transiently. However, at the age of 85 years, low back pain and left leg pain recurred, and he could not walk continuously. An MRI showed posterior spondylolisthesis, a bulging disc, and canal stenosis at L3-4. Additional fusion surgery at L2-3 was performed for proximal adjacent segment disease (ASD) (Figure 2). His leg pain decreased after reoperation. Nevertheless, he had severe locomotive dysfunction even though after conventional physical rehabilitation.

### 2.2. Exercise Therapy Using the Lumbar-Type HAL

**Figure 3:**
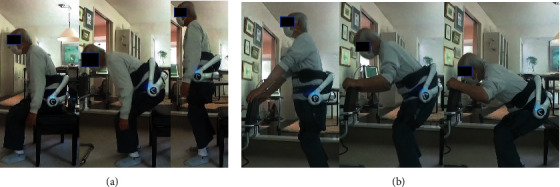
Exercise therapy using the lumbar-type HAL: (a) sit-to-stand training and (b) squat training.

Exercise therapy using the lumbar-type HAL was performed beginning 4 months after his final surgery. This was comprised of sit-to-stand training and squat training with the joint motion assistance of the lumbar-type HAL. First, he equipped the lumbar-type HAL. He began sit-to-stand training from the sitting position and stood up without hand support. The sit-to-stand motion was repeated 20 times in each set. Next, he squatted from the standing position while holding on to the chair placed in front of him. He bent his knee joint approximately 45 degrees (quarter squat) and extended the knee joint to the standing position. This patient performed 3 sets of 20 sit-to-stand and 20 squat repetitions with adequate rests in between sets (Figure 3). The HAL training was conducted every day for 12 weeks.

### 2.3. Measurement

Timed up and go test (TUG), 1-minute sit to stand test (1MSTS), one-leg stand test (OLST), and Berg balance scale (BBS) were assessed as locomotive function measurement. Before HAL therapy, TUG, 1MSTS, OLST, and BBS were 18.1 sec, 20 times, less than 1 sec, and 47, respectively. He could not walk without assistance. After the exercise therapy with the lumbar-type HAL, his locomotive function dramatically improved. TUG, 1MSTS, OLST, and BBS were 12.2 sec, 25 times, 3.9 sec, and 52, respectively. Moreover, the patient could walk 60 meters continuously without assistance.

## 3. Discussion

In this case, exercise therapy with the lumbar-type HAL was performed for locomotive dysfunction that remained after lumbar fusion surgery. This patient was able to perform sit-to-stand and squat training using HAL safely for 3 months. Accordingly, he obtained improvement in locomotive function. It seems that the lumbar-type HAL may contribute to his locomotive improvement by preventing lumbar overload and assisting voluntary hip joint motion.

Madera et al. [6] reported that various options such as cardiovascular exercise, soft-tissue mobilization, nerve mobilization, motor control, strength training, joint mobilization, and patient education have been suggested for rehabilitation after lumbar fusion surgery. In addition, Greenwood et al. [12] published a systematic review and meta-analysis that suggested “complex rehabilitation” comprising exercise and cognitive behavioral therapy could offer functional benefits to patients following LFS; however, a lack of high quality research regarding established rehabilitation protocols remained. Optimal rehabilitation intervention remains controversial.

This case indicates that exercise therapy using the lumbar-type HAL may especially contribute to improvements in locomotive functional after LFS. Watanabe et al. [13] suggested the lumbar-type HAL might reduce the cardiopulmonary burden during stand-up or squat exercises and help a wearer increase the frequency of these exercises. We believe that overload might be avoided during exercise by using the lumbar-type HAL in this case, resulting in sufficient training. Moreover, Kotani et al. [14] reported that motor functions evaluated by 10MWT and TUG improved after core exercises and squats with the use of the lumbar-type HAL in elderly patients with physical frailty. The lumbar-type HAL might be a treatment option for physical frailty even when complicated by spinal problems. Lumbar stiffness after LFS may be ameliorated with use of the lumbar-type HAL by suppressing lumbar overload and assisting with voluntary hip joint motion. To the best of our knowledge, this is the first case report of exercise therapy using the lumbar-type HAL after LFS. There may be a possibility of an effective rehabilitation option to improve locomotive function for patients with lumbar stiffness after LFS. However, the possibility cannot be excluded that only higher-intensity exercise could be effective regardless of the lumbar-type HAL. Besides, the mechanism of improvement has not been elucidated. For these reasons, further studies of postoperative rehabilitation using the lumbar-type HAL following LFS are needed.

## 4. Conclusion

This elderly patient with lumbar stiffness after multiple lumbar fusion surgeries could perform sit-to-stand and squat training using the lumbar-type HAL safely without an adverse event, resulting in improvement in locomotive function. The unique characteristics of the lumbar-type HAL to prevent the lumbar overload and assist the voluntary hip joint motion during exercise therapy may be effective for lumbar stiffness. Sit-to-stand training and squat training using the lumbar-type HAL are promising options to improve locomotive function in elderly patients after lumbar fusion surgery.

## Data Availability

The datasets used and/or analyzed in this study are available from the corresponding authors on reasonable request.
